# Retraction: Radical Covalent Organic Frameworks Associated with Liquid Na‐K toward Dendrite‐Free Alkali Metal Anodes

**DOI:** 10.1002/advs.202302383

**Published:** 2023-05-26

**Authors:** Jianyi Wang, Menghui Chen, Zicong Lu, Zhida Chen, Liping Si

## Abstract

10.1002/advs.202203058

*Adv. Sci*. **2022**, *9*, 2203058

The above article from *Advanced Science*, published online on 21 July 2022 in Wiley Online Library (https://onlinelibrary.wiley.com/doi/full/10.1002/advs.202203058), has been retracted by agreement between the authors, the Editor‐in‐Chief, Kirsten Severing, and Wiley‐VCH GmbH. The retraction has been agreed as the article is based on research results and data that the authors were not authorized to use. Moreover, the majority of co‐authors have been listed despite insufficient qualification for contributorship.

10.1002/advs.202203058

*Adv. Sci*. **2022**, *9*, 2203058

The above article from *Advanced Science*, published online on 21 July 2022 in Wiley Online Library (https://onlinelibrary.wiley.com/doi/full/10.1002/advs.202203058), has been retracted by agreement between the authors, the Editor‐in‐Chief, Kirsten Severing, and Wiley‐VCH GmbH. The retraction has been agreed as the article is based on research results and data that the authors were not authorized to use. Moreover, the majority of co‐authors have been listed despite insufficient qualification for contributorship.

